# Genome editing in the mouse brain with minimally immunogenic Cas9 RNPs

**DOI:** 10.1016/j.ymthe.2023.06.019

**Published:** 2023-07-04

**Authors:** Elizabeth C. Stahl, Jennifer K. Sabo, Min Hyung Kang, Ryan Allen, Elizabeth Applegate, Shin Eui Kim, Yoonjin Kwon, Anmol Seth, Nicholas Lemus, Viviana Salinas-Rios, Katarzyna M. Soczek, Marena Trinidad, Linda T. Vo, Chris Jeans, Anna Wozniak, Timothy Morris, Athen Kimberlin, Thomas Foti, David F. Savage, Jennifer A. Doudna

**Affiliations:** 1 Innovative Genomics Institute, University of California, Berkeley, Berkeley, CA 94720, USA; 2 California Institute for Quantitative Biosciences (QB3), University of California, Berkeley, Berkeley, CA 94720, USA; 3 Department of Molecular and Cell Biology, University of California, Berkeley, Berkeley, CA 94720, USA; 4 Aldevron LLC, Madison, WI 53719, USA; 5 Howard Hughes Medical Institute, University of California, Berkeley, Berkeley, CA 94720, USA; 6 Department of Chemistry, University of California, Berkeley, Berkeley, CA 94720, USA; 7 MBIB Division, Lawrence Berkeley National Laboratory, Berkeley, CA 94720, USA; 8 Gladstone Institutes, University of California, Berkeley, San Francisco, CA 94114, USA

**Keywords:** CRISPR-Cas9, genome editing, viral vectors, non-viral delivery, mouse, brain, host immune response, neurons, microglia, endotoxin/LPS

## Abstract

Transient delivery of CRISPR-Cas9 ribonucleoproteins (RNPs) into the central nervous system (CNS) for therapeutic genome editing could avoid limitations of viral vector-based delivery including cargo capacity, immunogenicity, and cost. Here, we tested the ability of cell-penetrant Cas9 RNPs to edit the mouse striatum when introduced using a convection-enhanced delivery system. These transient Cas9 RNPs showed comparable editing of neurons and reduced adaptive immune responses relative to one formulation of Cas9 delivered using AAV serotype 9. The production of ultra-low endotoxin Cas9 protein manufactured at scale further improved innate immunity. We conclude that injection-based delivery of minimally immunogenic CRISPR genome editing RNPs into the CNS provides a valuable alternative to virus-mediated genome editing.

## Graphical abstract

**Figure:**
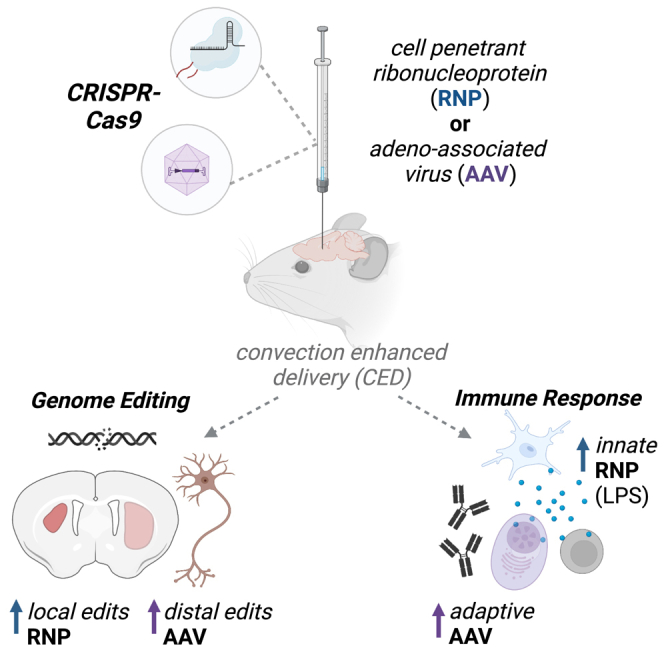

## Teaser

Doudna and colleagues show that cell-penetrant CRISPR-Cas9 ribonucleoproteins (RNPs) enable significant genome editing in neurons with a convection-enhanced delivery system. The transient, low endotoxin Cas9-RNPs also limit immune responses in the brain and periphery. These experiments demonstrate the potential of non-viral delivery of CRISPR-Cas9 for future therapeutic applications.

## Introduction

Editing somatic cells directly *in vivo* is anticipated to be the next wave of therapeutics for many genetic diseases, especially those affecting the central nervous system (CNS).^1^^,^^2^ Clustered regularly interspaced short palindromic repeats (CRISPR) is a revolutionary tool adapted from bacterial immune systems for genome editing.^3^^,^^4^^,^^5^ To achieve gene disruption, the functional endonuclease, Cas9, is directed by a guide RNA to a target site in DNA to generate a double-strand break leading to insertions and deletions (indels). Unfortunately, despite many genetic disease indications, the brain remains a challenging target for genome editing.

To circumvent the blood-brain barrier (BBB), most genomic medicines rely on direct intracranial injection of viral vectors encoding the transgene of interest. Viral vectors, such as recombinant adeno-associated virus (AAV), have had great success in gene therapy and are less immunogenic than most viral vectors; however, they require re-manufacturing for each target and are hindered by costly production scale-up. In addition, AAV has a limited DNA packaging capacity, and is associated with immunogenicity in the brain from both the vector and expression of foreign transgenes.^6^^,^^7^^,^^8^^,^^9^ Although the brain has been considered an immune-privileged site, green fluorescent protein can induce a strong inflammatory response and neuronal cell death 3 weeks after injection with AAV serotype 9 has been reported.^6^^,^^7^^,^^8^^,^^9^ In addition, Cas9-specific immune responses have been elicited following AAV delivery in mice^10^^,^^11^^,^^12^ and pre-existing cellular and humoral immunity to Cas9 and AAVs are documented in humans.^13^^,^^14^^,^^15^^,^^16^^,^^17^^,^^18^ Despite these drawbacks, AAVs are the most clinically relevant delivery systems currently in use for the CNS.

The development of transient, non-viral delivery systems that can effectively edit neurons throughout the brain with minimal immunogenicity would greatly facilitate future clinical applications. Previously, we developed cell-penetrating Cas9 ribonucleoproteins (RNPs) capable of genome editing in mouse neurons both *in vitro* and *in vivo*.^19^ To enable self-delivery of the Cas9 RNPs, four repeats of the positively charged Simian vacuolating virus 40 nuclear localization sequences (SV40-NLS) were fused to the N terminus along with two repeats to the C terminus of Cas9, a strategy that was also reported to enable delivery of zinc-finger nucleases.^20^ Using a single guide to turn on the tdTomato reporter from the lox-stop-lox Ai9 mouse,^21^ we reported edited striatal volume of approximately 1.5 mm.^3^^,^^19^

Here, we report further optimization of cell-penetrant Cas9 RNPs, demonstrating efficacy in human primary cells and improved editing of the mouse striatum using a convection-enhanced delivery (CED) system. We compared the transient RNP complexes with AAV serotype 9 for Cas9 delivery to the CNS, to measure both editing efficiency and the host immune response. We found that the Cas9-AAV was able to better diffuse throughout the brain, leading to distally edited cells; while the cell-penetrant Cas9-RNPs edited more neurons within the region near the injection site. Both groups elicited humoral responses, but vehicle-specific antibodies in the Cas9-AAV group persisted at higher levels out to 90 days. Cas9-AAV-treated brains were also associated with significantly elevated *Cd3e* gene expression at 4 weeks, suggesting an ongoing adaptive immune response. Cas9-RNP-treated brains showed acute microglial activation that was mitigated by reducing endotoxin levels during protein manufacturing scale-up. Taken together, Cas9 RNPs are a promising strategy for future therapeutic intervention in neurological disorders to address current limitations of viral delivery.

## Results

### Development of Cas9 cell-penetrant RNP and AAV to measure genome editing with the tdTomato reporter system

**Figure 1:**
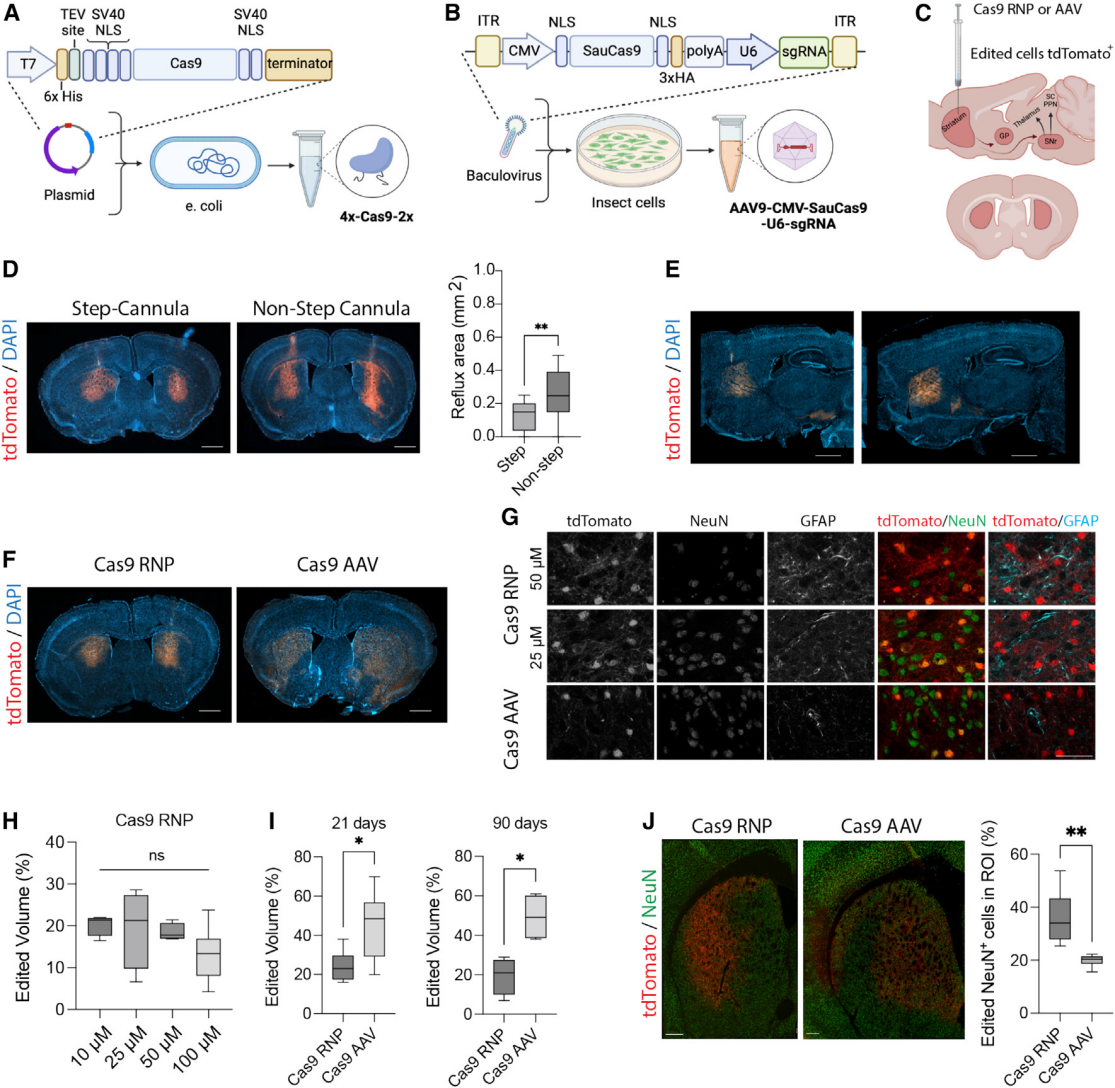
*In vivo* editing at tdTomato locus with viral and non-viral Cas9 delivery strategies (A) Schematic of 4x-SpyCas9-2x cell-penetrant protein expression and purification systems, (B) AAV9-SauCas9-sgRNA expression and purification systems, and (C) expected edited brain regions in the basal ganglia shown in sagittal view (top) and coronal view (bottom). Striatal neurons extend into the globus pallidus and substantia nigra. Created with BioRender.com. (D) Comparison of convection-enhanced delivery (CED) of cell-penetrant 4x-SpyCas9-2x RNP with step and non-step cannulas (n = 3–6 injections per group, unpaired t test, ∗∗p < 0.01.) Scale bars, 1 mm. (E) Serial sections of single hemisphere sagittal view of edited tdTomato^+^ cells in the basal ganglia circuit after injection of Cas9 RNP with CED into the striatum. Scale bar, 1 mm. (F) Representative coronal section of the striatum of mice that received Cas9 RNPs and AAVs at 21 days post-injection, showing the distribution of tdTomato^+^ edited cells. Scale bar, 1 mm. (G) Co-staining of tdTomato with NeuN and GFAP in the striatum at 90 days post-injection. Scale bar, 50 μm. (H) Volume of edited striatal tissue as the concentration of injected Cas9 RNPs was increased from 10 to 100 μM (n = 4–6 injections, one-way ANOVA, ns). (I) Quantification of editing following treatment with Cas9 AAV (3e-9 vg/μL, 1.5e-10 vg/hemisphere) and Cas9 RNPs (25 μM, 125 pmol/hemisphere) at 21 and 90 days (n = 4–6 injections, one-way ANOVA, ∗p < 0.05). (J) Co-expression of tdTomato and NeuN quantified per regions of interest (ROIs), e.g., edited area per hemisphere (n = 4–6 injections, one-way ANOVA, ∗∗p < 0.01). Scale bars, 250 μm.

Creating a large deletion in the lox-stop-lox cassette in Ai9 mice with a single-guide RNA (sgRNA) enables expression of tdTomato and efficient quantification of editing by fluorescent readout (Figure S1A). Cas9 from *Streptococcus pyogenes* (engineered with four copies of SV40 NLS on the N terminus and two copies on the C terminus [4x-SpyCas9-2x] to be cell penetrant) was first produced from recombinant *E. coli* in a laboratory setting, using a low endotoxin method. Editing efficiency of the RNP was compared with Cas9 delivered by recombinant AAV (Figures 1A–1C). Since SpyCas9 cannot be packaged within a single AAV with its guide RNA, we used clinically relevant AAV-SauCas9-sgRNA (derived from *Staphylococcus aureus*).^22^^,^^23^^,^^24^ AAV serotype 9 was produced using a baculovirus transfected into Sf9 insect cells.^25^^,^^26^ To control for differences in the Cas9 orthologs, cell-penetrant 4x-SauCas9-2x protein was also produced following the same expression and purification methods as 4x-SpyCas9-2x. Due to differences in PAM requirements between the two Cas9 orthologs (SpyCas9 NGG and SauCas9 NNGRRT), a new guide was designed for SauCas9 to target the tdTomato locus (Figure S1B).

We confirmed editing in neural precursor cells (NPCs) isolated from embryonic day 13.5 Ai9 mice with all constructs *in vitro* (Figure S1). Addition of 4x-SauCas9-2x RNP into the cell culture supernatant enabled editing of NPCs compared with 0x-SauCas9-2x RNPs, demonstrating that the four SV40-NLS peptides can mediate delivery of additional Cas9 orthologs. 4x-SauCas9-2x RNPs were slightly less efficacious than 4x-SpyCas9-2x RNPs, which could be explained in part by differences in the guide RNAs (Figures S1A and S1B). We observed editing of mouse NPCs with SauCas9 when delivered as both cell-penetrant RNP (59% ± 6% tdTomato cells^+^, Figures S1C and S1D) and AAV (42% ± 1% tdTomato cells^+^, Figure S1E). The same titer of AAV9-CMV-GFP resulted in 96% ± 2% GFP^+^ cells, suggesting that editing lagged behind transduction; and an increase in empty capsids in the AAV-CMV-SauCas9 group was noted (Figure S1F).^27^^,^^28^

To further examine the potential for cell-penetrant Cas9 RNPs to edit difficult cells *in vitro*, we tested delivery and editing with 4x-SpyCas9-2x in human NPCs derived from induced pluripotent stem cells (iPSCs).^29^^,^^30^^,^^31^ Human NPCs were treated with pre-formed RNPs using an established guide RNA targeting EMX1.^32^ In human cells, we detected 10-fold higher rates of editing with 4x-SpyCas9-2x compared with standard RNPs (0x-SpyCas9-2x) delivered with commercial transfection reagents (Figures S2A and S2B).

### Cas9 RNPs result in modest editing of brain parenchyma following delivery into cerebrospinal fluid

To determine the optimal route of delivery for Cas9 RNPs into the mouse CNS, we tested intraparenchymal injections into the striatum, as well as injection into the cerebrospinal fluid (CSF), including intrathecal (i.t.) and intracerebroventricular (i.c.v.) routes. Following i.t. injection of cell-penetrant RNPs, we observed edited glial cells and neurons in the cortex and striatum of one hemisphere, but no editing within the spinal cord (Figure S3A). Following i.c.v. injection of Cas9 RNPs in neonatal p0 mice, we observed tdTomato^+^ cells in the subventricular zone and white matter, including glial cells and neural stem/progenitor cells expressing Ki67 and DCX evaluated 3 weeks after delivery (Figures S3B and S3C). Editing post-i.c.v. injection in adult mice was restricted to the cells within the lateral ventricles, choroid plexus, subventricular zone, and hippocampus in a subset of mice (Figures S3D and S3E). The total number of edited cells with RNP delivery into the CSF was lower than with direct intraparenchymal injection (Figure S3F).

Therefore, we sought to further improve upon intraparenchymal injections using a CED system, which generates a high-pressure gradient to aid in biodistribution of macromolecules in the brain. CED has been used to increase the spread of AAV in the brains of large animal models and humans by infusing relatively high injection volumes at high rates.^33^^,^^34^ In addition, we tested two needle designs to enable CED with Cas9-RNPs. We found that the CED enabled robust editing in the mouse striatum (Figure S4) and the step-cannula reduced reflux of RNP from the needle-injection track, as reported previously^35^ (Figure 1D). Furthermore, tdTomato^+^ neurons edited by Cas9-RNPs within the striatum were observed to extend along the basal ganglia circuit into the globus pallidus and substantia nigra along the anterior-posterior axis (Figure 1E).

### CED of Cas9 RNPs and AAVs mediates robust editing in the mouse striatum

Using bilateral CED injections into the striatum, we compared edited tissue volume with the 4x-SpyCas9-2x RNP, 4x-SauCas9-2x RNP, and AAV9-SauCas9-sgRNA in adult Ai9 mice at 3 weeks post-injection. Despite performing well *in vitro*, 4x-SauCas9-2x RNPs underperformed *in vivo* when tested at two different doses and additional NLS configurations (Figure S5). Therefore, we performed our primary comparison with two orthologous systems: 4x-SpyCas9-2x RNP (hereafter referred to as Cas9-RNP) and AAV9-SauCas9-sgRNA (hereafter referred to as Cas9-AAV, which serves as a positive control, Figure 1F).

First, we tested Cas9-AAV injection with CED at two doses, 3 × 10^8^ and 3 × 10^9^ vg/μL (1.5 × 10^9^ to 1.5 × 10^10^ vg/hemisphere),^36^ and proceeded with the higher dose for subsequent studies *in vivo* (Figures S6A and S6B). We also tested several doses of Cas9-RNP ranging from 10 to 100 μM (50–500 pmol/hemisphere). Interestingly, there was no significant difference in editing when delivering RNPs in this concentration range (Figures 1H and S6C). We chose the 25-μM RNP concentration (4.15 mg/mL or approximately 1.75 mg/kg Cas9) group for further study as it had the highest maximal editing rate. Above 25μM in the RNP group, we observed a decrease in NeuN staining and an increase in GFAP staining out to 90 days in the Cas9-RNP group, suggesting dose-limiting effects (Figure 1G).

At both 21 and 90 days post-injection, the Cas9-AAV group outperformed the Cas9-RNP group when quantifying total edited striatal volume (n = 8 at 21 days, n = 4 at 90 days, p < 0.05, Figure 1I). The volume of edited cells was relatively stable in the Cas9-AAV group between 21 and 90 days at approximately 47% ± 3% (covering approximately 13.4 mm^3^ of striatum), while the Cas9-RNP group had editing levels of 22% ± 3% (approximately 6.2 mm^3^ of striatum) between 21 and 90 days (increased from previous report of editing 1.5 mm^3^ striatal volume^19^). Edited cells were observed further along the rostral-caudal axis in the Cas9-AAV group (−2.12–2.5 mm relative to bregma), demonstrating better diffusion of the editor away from the injection site (Figures S6E and S6F).

Since large deletions in the tdTomato locus make on-target editing difficult to assess using short-read next-generation sequencing (NGS), we developed an NHEJ droplet digital PCR (ddPCR) assay to measure drop-off of HEX-labeled probes over the cut sites in relation to distal reference FAM-labeled probes. Genomic DNA was isolated from 2-mm-thick sections of each injected hemisphere, covering multiple brain sub-structures. Loss of the HEX probe reached 2% ± 1% in the Cas9-RNP group and 15% ± 10% in the Cas9-AAV group, indicating edited alleles, when measured at 28 days (Figure S7).

We also quantified the percentage of edited NeuN^+^ neurons within the tdTomato^+^ region of interest (ROI) per hemisphere between Cas9-AAV and Cas9-RNP at the 21-day time point. We found that Cas9-RNP edited significantly more NeuN^+^ neurons per ROI (36% ± 10%) compared with Cas9-AAV (20% ± 2%) (Figure 1J, n = 6–8 injections, p < 0.05). Within the ROI, neurons were the most frequently edited cell type in both groups, including DARPP-32^+^ medium spiny neurons (Figure S8). In addition, editing of ALDH1L1^+^ and OLIG2^+^ glial cells was noted in both groups (approximately 2% of edited cells within the ROI in the Cas9-RNP group and 8% of cells in the Cas9-AAV group). Therefore, Cas9-RNPs were able to edit comparable numbers of neurons and glia as Cas9-AAVs within a given area of striatal tissue.

### Comparison of local and peripheral immune response between Cas9 RNPs and AAVs in the Ai9 reporter mouse

**Figure 2:**
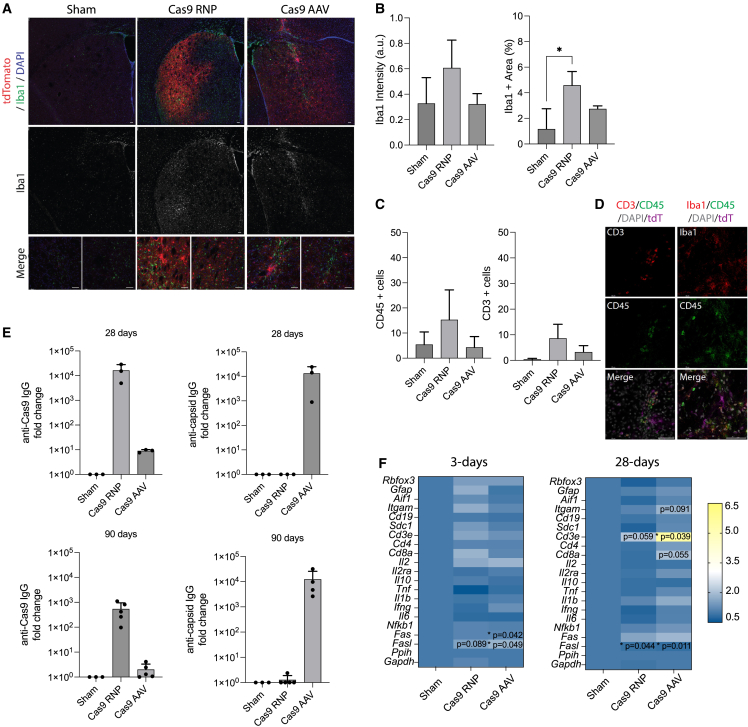
Immune response following *in vivo* editing with viral and non-viral Cas9 delivery strategies (A) Representative immunostaining of Iba1 (microglia, green) with tdTomato and DAPI using confocal microscopy. Scale bar, 50 μm. (B) Quantification of Iba1^+^ staining intensity and percent area (n = 4–6 technical replicates, one-way ANOVA, ∗p < 0.05). (C) Quantification of CD45^+^ and CD3^+^ cells per image (n = 3–6 replicates, one-way ANOVA, ns). (D) Representative images of CD45, CD3, and Iba1 showing co-expression of CD45 (green) with both Iba1 (microglia, red) and CD3 (T cells, red) cells and differential cell morphology. Merged images include DAPI (gray) and tdTomato (magenta). Scale bars, 50 μm. (E) Quantification of IgG antibodies against Cas9 or AAV capsid proteins measured 28 and 90 days after bilateral intrastriatal injections by ELISA (n = 3–5 biological replicates). (F) Heatmap summarizing qRT-PCR results of gene expression from homogenized brain tissue near the injection site (striatum and cortex) at two time points. *Ppih* was used as a housekeeping control for delta-delta Ct analysis and compared with the sham group using the QIAGEN analysis portal (n = 4, ∗p < 0.05).

We next examined the local and peripheral immune response following delivery of Cas9 RNPs and AAVs into the brain. Using immunofluorescent staining for Iba1 (Figure 2A), we observed a significant increase in percent Iba1^+^ area in the 25-μM Cas9-RNP group from sham-treated animals (Figure 2B, n = 6 replicates, p < 0.05). Staining for CD45 showed co-expression on Iba1^+^ microglia and CD3^+^ T cells, which were slightly increased in the 25 μM Cas9-RNP group compared with the sham and Cas9-AAV, but not significantly different, at 3 weeks post-injection (Figures 2C and 2D, n = 6–12 replicates).

In addition to the immune response at the local site of injection, circulating IgG antibodies were measured at 28 and 90 days post-injection. We found that sham-treated animals had no pre-existing antibodies to SpyCas9, SauCas9, or AAV9 capsids. At 28 days after striatal injection, there was a 1.6e4-fold increase in anti-SpyCas9 IgG in the 25-μM Cas9-RNP group, a 1.3e4-fold increase in anti-AAV9 capsid IgG in the Cas9-AAV group, and an 8.9e1-fold increase in anti-SauCas9 IgG in the Cas9-AAV group (i.e., humoral response against expressed transgene) (Figure 2E, n = 3–5 biological replicates). No cross-reactivity was observed between ortholog RNPs, as described previously,^11^ nor were any anti-AAV capsid antibodies detected in the RNP group. At 90 days, the levels of IgG fell to a 5.4e2-fold increase in the 25-μM Cas9 RNP group and 1.2e4-fold increase in the Cas9 AAV group from the sham controls, demonstrating greater maintenance of systemic antibodies against the capsid in the AAV group.

The cellular and humoral immune response to Cas9 RNPs was dose dependent and a significant increase in CD45^+^ cells was observed at the 100-μM RNP dose compared with sham, Cas9-AAV, and Cas9-RNP at 25 μM (Figures S9A–S9D). Cas9-reactive cells were also identified in the spleen by interferon-gamma (IFN-γ) enzyme-linked immunospot (ELISpot) assay (Figure S9E) at both 25- and 100-μM doses of Cas9-RNPs, but not in sham-treated animals.

Since the mice had no pre-existing antibodies to SpyCas9, we tested how the immune response would differ in the RNP group by first exposing the mice to a single subcutaneous injection of 4x-SpyCas9-2x protein with adjuvant (AddaVax) 4 weeks before stereotaxic surgery with Cas9-RNPs. We found that pre-exposing the mice to Cas9 and adjuvant had a synergistic effect on both serum IgG and activation of IFN-γ^+^ cells in the spleen (Figures S9F–S9I). Mice that received surgery maintained tdTomato^+^ cells in the brain to the measured time point. Additional studies using this immunization strategy may help to further characterize the immune response to Cas9-RNPs.

Finally, we measured gene expression changes near the injection site in mice that received Cas9-RNP and AAV at 3 and 28 days post-injection using qRT-PCR. At 3 days, the Cas9-AAV group had a modest but significant increase in *Fas* (1.19-fold) and *Fasl* (1.85-fold) compared with the sham group (Figure 2F, n = 4 replicates, p < 0.05). At 28 days post-injection, both Cas9-RNP and -AAV had a significant increase in *Fas* (1.61- and 1.89-fold, respectively). In addition, the Cas9-AAV group had a significant increase in *Cd3e* gene expression (5.45-fold, p = 0.039), closely followed by *Cd8a* (2.06-fold, p = 0.055), while Cas9-RNP had a slight but non-significant increase in *Cd3e* (2.15-fold, ns, p = 0.059) compared with the sham group.

There were no detectable off-target editing events at 1 and 4 months post-injection in any of the experimental groups at the evaluated sites (Figures S10A–S10C). In the Cas9-AAV group, the Cas9 transgene was expressed in the brain out to 4 months, the last tested time point, as expected (Figure S10D). In addition, few genes were differentially expressed between the groups at 4 months, except for *Fas* (1.54-fold, p < 0.05), which was significantly elevated in the Cas9-AAV group compared with the sham (Figures S10E and S10F). We used long-read sequencing to examine whether any fragments of the viral genome had been integrated near the cut site in the tdTomato locus, as reported previously.^37^^,^^38^^,^^39^^,^^40^ We also observed partial integrations of viral fragments in our amplicon, although our *in vivo* editing rates and sequencing depth were relatively low (Figure S11).

Overall, delivery of Cas9 by either AAV or RNP resulted in activation of immune responses in the brain and periphery, although with generally small effect sizes compared with sham-injected mice. The increased Iba1^+^ cells near tdTomato^+^ cells in the striatum of the 25-μM Cas9-RNP group raised the question of whether the response was due to Cas9 itself or impurities within the protein product. We hypothesized that the local immune response may be due to endotoxins from *E. coli* in the RNP complexes.

### Production and testing of ultra-low endotoxin 4x-SpyCas9-2x protein

**Figure 3:**
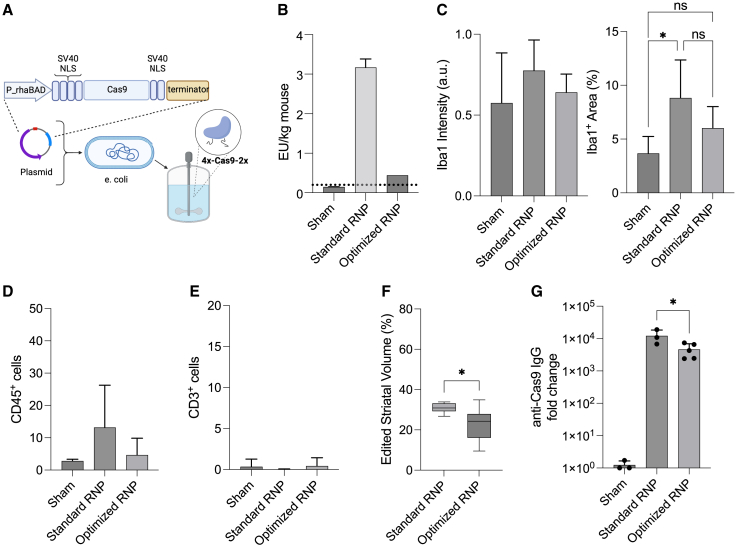
Optimized, low endotoxin RNP formulation reduces local immune response (A) Schematic of manufacturing scale-up to produce industrial ultra-low endotoxin 4x-SpyCas9-2x protein using a tag-free expression and purification system. (B) Endotoxin levels calculated on a per mouse basis between the standard (laboratory 4x-SpyCas9-2x with sg298 2018) and optimized (industrial 4x-SpyCas9-2x protein with sg298 2022) RNP formulations at 25 μM measured by LAL assay. Dotted line indicates FDA recommendation of 0.2 EU/kg/h for drug products administered intrathecally in humans. (C) Quantification of Iba1^+^ staining intensity and percent area (n = 6–10, one-way ANOVA, ∗p < 0.05). (D) Quantification of CD45^+^ and (E) CD3^+^ cells per image (n = 6–10, one-way ANOVA, ns). (F) Percent volume of edited striatal tissue for Cas9 RNPs injected at 25 μM (n = 6–10 injections). (G) Quantification of IgG antibodies against Cas9 or AAV capsid proteins measured 21 days after bilateral intrastriatal injections by ELISA (n = 3–5 biological replicates).

To examine the impact of endotoxin on the immune response to RNPs, we partnered with a commercial producer of Cas9 protein and were able to significantly scale-up manufacturing to produce a large quantity of ultra-low endotoxin 4x-SpyCas9-2x protein using an industrial tag-free expression and purification system (Figure 3A).

Using the limulus amebocyte lysate (LAL) assay, we measured an endotoxin concentration of 0.035 EU/mg in the industrial-produced protein compared with 0.2 EU/mg in the lab-produced protein (Figure S12). Interestingly, using the same assay, we found that guide RNA could be an unexpected source of endotoxin contamination. Endotoxin was present in at least three unopened vials of lyophilized RNA that had been stored at −80°C from a 2018 lot, but not in a more recently purchased lot from the same vendor when resuspended simultaneously (Figures S12A–S12C). To rule out false positives due to reaction of LAL with beta-glucans, we performed the recombinant factor C (rFC) assay. We measured a similar level of endotoxins in the guides between the LAL and rFC assays, demonstrating the positive signal was from contamination with endotoxin and not beta-glucans (Figures S12D and S12E). Furthermore, the amount of endotoxin increased with dose of RNP and was substantially reduced with the new guide and protein, as expected (Figures S12F and S12G).

To measure the physiological impact of endotoxin in our samples, we used HEK293 cells that were engineered to produce secreted embryonic alkaline phosphatase (SEAP) downstream of NF-κB activation resulting from human Toll-like receptor 4 (hTLR4) stimulation with endotoxin/lipopolysaccharide (LPS) (Figures S13A–S13C). The lab-produced protein stimulated NF-κB in HEK293 cells significantly greater than the industrial produced protein (Figure S13D, p < 0.01, unpaired t test). Treatment with the industrially produced protein led to similar levels of SEAP between hTLR4 cells and the parental cell line (Null2), demonstrating that most of the NF-κB stimulation was downstream of other pattern-recognition receptors (such as TLR3, TLR5, or nucleotide-binding oligomerization domain-containing protein 1 activation) and not due to LPS signaling through hTLR4. When combined with sg298 from the 2018 or 2022 lots, absorbance levels of SEAP further increased in RNP complexes made with lab-produced protein, while the industrial protein with either guide did not induce a response (Figure S13E). Furthermore, guide RNA alone did not stimulate NF-κB in HEK293 cells (Figure S13F).

Finally, we measured endotoxins in the “optimized” formulation of RNPs, comprised of the industrially produced 4x-SpyCas9-2x protein and 2022 lot of sgRNA, using the LAL assay. Estimating delivery of 10 μL per mouse, the endotoxin burden was 0.44 EU/kg when RNPs were formulated at 25 μM. These data suggest that RNPs could be delivered below the 0.2-EU/kg FDA threshold for i.t. delivery^41^ when formulated at 10 μM without significant loss of editing (Figures 3B and 1H).

### Optimized RNP formulation reduces immune response

We performed CED bilateral intrastriatal injections to test if reducing endotoxins would improve the host immune response to RNPs *in vivo*. In this experiment, we compared the optimized RNP formulation (industrially produced 4x-SpyCas9-2x NLS protein with sg298 2022) to the standard formulation used in Figures 1 and 2 (laboratory produced 4x-SpyCas9-2x NLS protein with sg298 2018) at 25 μM. The standard RNP induced a significant increase in Iba1^+^ area, consistent with our previous measurements (Figures 3C and 1A); however, the optimized Cas9-RNP formulation did not induce microglial activation. In addition, there was no increase in CD45^+^ and CD3^+^ cells from the sham in the optimized RNP group (Figure 3D). Of note, the standard RNP edited an average of 31% ± 3% striatal volume (greater than values reported in Figure 1, possibly due to differences between protein lots or injections), while the optimized RNP edited an average of 23% ± 8% striatal volume (Figure 3F). When tested *in vitro,* the optimized RNP performed slightly better at direct delivery than nucleofection compared with the standard formulation, which could explain in part the differences *in vivo* (Figure S14A). Interestingly, the standard RNP also led to significantly greater anti-Cas9 IgG responses at 3 weeks post-injection, possibly due to endotoxin boosting the adaptive immune response (Figure 3G). Taken together, we found that reducing endotoxins in both the guide RNA and protein components of the RNP leads to a reduced innate immune response, comparable with the sham, while maintaining high on-target editing. Furthermore, cell-penetrant Cas9 proteins are amenable to fast and cost-effective manufacturing of large quantities suitable for *in vivo* experiments.

In conclusion, our results establish complementary genome editing and immunogenicity outcomes between the two tested Cas9 delivery strategies (Figure S14B). To enable high levels of editing in neurons within a localized brain region, minimizing adaptive immune responses, and timely and affordable manufacturing scale-up, the RNP offers an effective alternative delivery system to viral vectors.

## Discussion

In this study, we demonstrate that cell-penetrant Cas9 RNPs edit a significant volume of the mouse striatum using CED. Furthermore, the 4x-NLS modification enables self-delivery of multiple Cas9 orthologs to neuronal cells from both mouse and human species. We also show that Cas9 RNPs have dose-dependent effects on the immune response, which can be mitigated by using ultra-low endotoxin protein produced in an industrial non-GMP setting. These experiments are informative for the design of future therapeutic applications of Cas9 RNP editors in mice and larger animal models.

Several studies have reported non-viral delivery of Cas9 into the mouse brain. The “CRISPR-Gold” Cas9 nanoparticle delivery system induced 14% edited glial cells near the injection site, sufficient to reduce repetitive behaviors in a mouse model of fragile X syndrome.^42^ In addition, incubating RNPs with R7L10, an arginine- and leucine-rich cationic peptide, induced 45% indels in the CA3 region of the hippocampus, leading to behavioral improvements in an Alzheimer’s disease mouse model.^43^ Efficient editing of DARPP-32 medium spiny neurons in the striatum was achieved here and in recent work by others using RNPs packaged in biodegradable PEGylated nanocapsules.^44^ Interestingly the PEGylated nanocapsules have a neutral charge, while 4x-Cas9-2x NLS RNPs have a net-positive charge, suggesting that the mechanism of entry may differ between the two strategies. Systemic delivery of genome editors with glucose-conjugated silica nanoparticles and AAV9 can also lead to modest levels of editing in the brain, sufficient for therapeutic benefit.^45^^,^^46^ Despite the need for direct injection, the simplicity of the 4x-Cas9-2x RNP is ideal from a manufacturing perspective compared with other formulations. In the future, the RNP injection buffer could be further supplemented with polymers, such as polyethylene glycol,^47^^,^^48^ to potentially improve distribution in the brain.

Several studies show correlation between editing at the tdTomato locus and subsequent editing at endogenous sites^42^^,^^45^^,^^49^ and future work will focus on genome editing at therapeutically relevant targets. It is important to note that expression of the tdTomato protein in the Ai9 mouse model underreports the actual genome editing efficiency, as double-strand breaks that result in indels and small deletions are not sufficient to turn on the reporter.^19^ We sought to further resolve editing outcomes with a ddPCR assay, since the tdTomato locus is too large for Illumina-based NGS. In the ddPCR probe drop-off assay we detected approximately a 6-fold difference in editing with AAV compared with RNP (approximately 15% vs. 2.5% indels), whereas our immunofluorescent measurement showed a 2-fold increase. We speculate that the difference in reported editing efficiency between the two assays is based on the sampling methodology. The image analysis workflow quantified the volume of striatal tissue containing edited cells (Figure 1I), where 20%–36% of neurons were edited within 25%–50% of the striatum (Figure 1J). Therefore, the image quantification reflects the maximal distribution of edited cells in the striatum. The ddPCR assay utilized genomic DNA (gDNA) from 2-mm-thick tissues dissected from the expected injection site including the striatum, as well as the cortex and corpus callosum, which likely favors editing events in the Cas9-AAV group due to its enhanced diffusion through the brain. While dose escalating the RNP did not improve editing levels, suggesting a saturating dose was already achieved as measured by image quantification, editing at different doses was not assessed using ddPCR. Furthermore, increasing the dose of Cas9-AAV may have further increased editing levels. Studies suggest that correcting pathological mutations in 20%–30% of striatal neurons expressing mutant huntingtin protein is sufficient to significantly improve the disease pathology, therefore even modest editing levels in the striatum could enable therapeutic benefit.^50^ Ultimately, behavioral assays are needed to further determine therapeutic benefit of the RNP approach in a disease-relevant model.

We hypothesized that cell-penetrant Cas9-RNPs would be less immunogenic than Cas9-AAVs due to their transient expression. As the dose of 4x-SpyCas9-2x RNPs increased from 25 to 100 μM, there was an increase in CD45^+^ and GFAP^+^ cells, and a decrease in NeuN^+^ cells. As such, subsequent experiments were performed at 25 μM, which was well tolerated and resulted in similar levels of editing as the higher dose. The 25-μM Cas9-RNP dose led to lower levels of vehicle-specific antibodies by 90 days post-injection compared with Cas9-AAVs and did not upregulate gene signatures of T cells at 28 days as measured by qRT-PCR, supporting our hypothesis. By 90 days, the levels of anti-Cas9 antibodies in the Cas9-AAV group were only elevated in three out of five mice, demonstrating that the kinetics of Cas9 antibody persistence may be similar between RNPs and AAVs, despite stable, intracellular Cas9 expression. Reducing endotoxin in both the Cas9 protein and guide RNA prevented microglial reactions and reduced humoral responses at 21 days.

In the Cas9-AAV group, few immune cells (CD45, Iba1, or CD3) were observed in the striatum by immunostaining; however, *CD3e* gene expression was significantly upregulated in explanted tissue, closely followed by an increase in *CD8a*. This finding could indicate accumulation of cytotoxic T cells trafficking into the parenchyma from the blood vessels or CSF. In addition, no changes in NeuN, GFAP, and CD45 expression were observed in the Cas9-AAV group out to 4 months, demonstrating that the AAV delivery strategy was well tolerated overall in naive mice. A study by Li et al. found that mice immunized against SauCas9 with Freund’s adjuvant 1 week before intravenous delivery of AAV8-SauCas9-sgRNA resulted in accumulation of cytotoxic T cells in the liver and subsequent removal of edited hepatocytes.^12^ Therefore, the host immune response to Cas9-AAV in mice with pre-existing immunity would likely be different than what we observed in naive mice. In the Cas9-RNP group, we found that pre-exposing mice to SpyCas9 protein with AddaVax adjuvant 4 weeks before stereotaxic surgery synergistically increased systemic adaptive immune responses. Further studies are needed to assess the immune response to Cas9-AAV and RNP in models with pre-existing immunity, but how well these immunized mouse models recapitulate pre-existing immunity in humans is not clear. Furthermore, breakdown of the BBB in the context of neurodegenerative disease or strong expression of the tdTomato fluorescent reporter could also impact the host immune response.^51^^,^^52^

In this study, we used a strong CMV-promoter to drive expression of SauCas9 from the AAV, which allowed us to assess all subsets of edited cells in the striatum, in comparison with the RNP, which is not inherently designed to be neuron specific. Although the SauCas9 transgene was still expressed 4 months post-delivery, editing at predicted off-target sites was not detected. Further work to experimentally determine guide-specific off-target sites, such as Guide-Seq^53^ or Circle-Seq,^54^ was not performed. To prevent potential genotoxic side effects due to long-term Cas9 expression, we recommend applying additional safeguards, such as AAV self-inactivation strategies, and neuron-specific promoters, such as human synapsin 1, to increase cell specificity.^55^^,^^56^^,^^57^ While self-inactivating AAVs may improve safety, they may not be sufficient to prevent integration of the viral genome at the Cas9 cut site, which has been reported.^37^^,^^38^^,^^39^^,^^40^ Strategies to mitigate the host response to genome editors, including immunosuppressants and screening for pre-existing immunity before dosing, should also be implemented when translating *in vivo* editing to humans.^58^

While SpyCas9 is generally the most efficacious and widely used type II CRISPR protein to date, it is too large to be packaged in a single AAV with sgRNA. Therefore, in this study, we used AAV-CMV-SauCas9-U6-sgRNA as a positive control to benchmark delivery of RNPs, as similar AAV constructs have been used extensively in the literature and in clinical trials.^12^^,^^23^^,^^24^^,^^59^ *In vivo* delivery of 4x-SauCas9-2x RNP did not lead to significant editing; thus, most comparisons were performed between two orthologous genome editors and delivery systems with different NLS configurations, which may impact the interpretation of this work. *In vivo* editing with 4x-SauCas9-2x RNP was possibly hindered by the reduced thermostability of the protein, as reported previously.^60^ Employing Cas9 RNPs from a more thermostable organism could be advantageous in the future. Since RNPs are not restricted by cargo packaging, one advantage of the cell-penetrant RNP technology is the ability to use SpyCas9 for future therapeutic applications in the brain.

In conclusion, the cell-penetrant 4x-Cas9-2x NLS fusion protein enables simple and effective delivery of Cas9 RNPs into neuronal cells *in vitro* and *in vivo.* To our knowledge, this study is the first to examine the host immune response to Cas9 in the brain, benchmark an RNP delivery strategy against the gold standard for gene delivery in the CNS, and demonstrate feasibility of large-scale manufacturing of cell-penetrant Cas9 protein. Given that Cas9-RNPs excel at editing high levels of neurons within a localized region of the brain, this is a promising modality to characterize therapeutic benefit in disease models in the future.

## Materials and methods

### Plasmid construction

Guide spacer sequences were cloned into a plasmid encoding SauCas9 (pSTX8,pKLT7.1_SaCas9prot_SaCas9guide) as described previously. In brief, oligonucleotides encoding the spacers were custom synthesized (Integrated DNA Technologies [IDT], Coralville, IA) and phosphorylated by T4 polynucleotide kinase (New England Biolabs, Ipswich, MA) for 30 min at 37°C. Oligonucleotides were annealed for 5 min at 95°C, cooled to room temperature, and ligated into the BsmBI restriction sites of the plasmid. The following 23-nt spacer sequences were cloned into the plasmid (spo 1: TGGTATGGCTGATTATGATCCTC; spo2: TCCCCCTGAACCTGAAACATAAA; spo3: GATGAGTTTGGACAAACCACAAC; spo4: TCCAGACATGGATACATTGATAA; spo5: CTCATCAATGTATCTTATCATGT), and plasmids were used for editing in mouse NPCs *in vitro*. The best performing SauCas9 spacer (spo4: TCCAGACATGATAAGATACATTG) was then cloned into an AAV2 backbone plasmid. pX601-AAV-CMV::NLS-SaCas9-NLS-3xHA-bGHpA; U6::BsaI-sgRNA was a gift from Feng Zhang (Addgene plasmid no. 61591; http://n2t.net/addgene:61591; RRID:Addgene_61591). In brief, the plasmid was digested using BbsI and a pair of annealed oligos were cloned into the guide RNA destination site by Golden Gate assembly. For SpyCas9, the sg298 spacer sequence (5′ AAGTAAAACCTCTACAAATG) was used to target tdTomato.^19^ Correct construction of all plasmids was verified by Sanger sequencing (UC Berkeley DNA Sequencing Facility).

### Recombinant AAV production

The custom AAV9-CMV-61591-HA-Bgh vectors were produced at Virovek (Hayward, CA) in insect Sf9 cells by dual infection with rBV-inCap9-inRep-hr2 and rBV-CMV-61591-HA-Bgh. The AAV9-CMV-GFP vectors were produced by dual infection with rBVinCap9-inRep-hr2 and rBV-CMV-GFP. The vectors were purified through two rounds of cesium chloride (CsCl) ultracentrifugation. The CsCl was removed through buffer exchange with two PD-10 desalting columns. Viral titer (approximately 2e-13 vg/mL) and purity were confirmed by NanoDrop spectrophotometer, real-time PCR, and SDS-PAGE protein gel analysis. The vectors were passed through 0.2-μm sterilized filters, tested for endotoxins (<0.6 EU/mL), as well as baculovirus and Sf9 DNA contamination (not detected).

### Purification of low endotoxin proteins in a laboratory setting

Protein expression and purification was performed in the QB3 Macrolab at UC Berkeley using a custom low endotoxin workflow. In brief, the plasmid, 4xNLS-pMJ915v2 (Addgene plasmid no. 88917; http://n2t.net/addgene:88917; RRID:Addgene_88917), was transformed into *E. coli* Rosetta2(DE3)pLysS cells (Novagen) and an overnight culture was used to inoculate 1-L flasks (12–24 L total per batch). Cells were grown for approximately 3 h at 37°C then cooled to 16°C. At optical density (OD) of 0.8–0.9, cells were induced and harvested after 16–18 h growth. Cells were lysed by homogenization in a buffer containing 1 mM MgCl_2_ and benzonase (1:1,000) to help reduce viscosity and centrifuged to remove insoluble material. Purification by Ni affinity (10 mL Ni resin for every 6 L cell lysate) was performed, and the bound protein was washed with 10 column volumes of buffer containing 0.1% Triton X-114 at 4°C to reduce endotoxins. Tag removal with TEV protease (1:100) was performed overnight at 4°C, then heparin affinity was used to concentrate each batch of protein, which was then flash frozen and stored at −80°C. A Sephacryl S300 size-exclusion column and flow path were sanitized with 0.5 M NaOH overnight, then washed with up to 3 column volumes of buffer to rinse and equilibrate the system. Frozen samples were thawed, combined, and adjusted to 4.5 mL, and the protocol was performed for size exclusion. Samples were refrigerated overnight. Sanitation and size exclusion were repeated the next day to further improve purity. Peak fractions were pooled, concentrated to 40 μM, aliquoted at 50 μL, flash frozen in liquid nitrogen, and stored at −80°C in sterile, endotoxin-free buffer 1 (25 mM NaP [pH 7.25], 300 mM NaCl, 200 mM trehalose [Sigma-Aldrich, St. Louis, MO, no. T5251]). The final average protein yield was 1 mg per 1 L cells. Plasmids for 2xNLS-SauCas9-2xNLS, 3xNLS-SauCas9-2xNLS, and 4xNLS-SauCas9-2xNLS were created by deletion mutagenesis using the existing 4xNLS-SpyCas9-2xNLS construct as a template. Correct construction of all plasmids was verified by Sanger sequencing (UC Berkeley DNA Sequencing Facility).

### Purification of ultra-low endotoxin proteins in an industrial setting

4x-SpyCas9-2x NLS protein was manufactured according to Aldevron proprietary workflows for expression and purification of gene editing nucleases. In brief, the gene for 4x-SpyCas9-2x NLS was synthesized (ATUM, Sunnyvale, CA) and cloned into a pD881 expression vector (ATUM). Expression-ready plasmid DNA was transformed into *E. coli* BL21(DE3) (New England Biolabs) culture in animal-free TB medium. At the appropriate OD_600_, expression was induced with 2.0% (w/v) Rhamnose and growth culture was harvested by centrifugation. Cells were lysed via dual-pass high-pressure homogenization and clarified via centrifugation. The clarified lysate was purified via multi-step chromatography using standard/commercially available resins. In the final chromatography step, the product is eluted via step elution and pooled to maximize final protein purity and minimize endotoxin. Product was dialyzed into the final formulation buffer, underwent three exchanges of buffer, and was pooled into a sterile vessel for final filtration and dispensing. Product was evaluated for key quality attributes including endotoxin via PTS Endosafe assay (Charles River, Cambridge, MA).

### Quantification of endotoxins in Cas9 RNPs

Proteins, guide RNAs, and RNP complexes were subjected to several assays to quantify endotoxin burden according to the manufacturer’s instructions. Assays were performed with autoclaved or certified pyrogen-free plasticware and endotoxin (ET)-free water. The plate-reader-based LAL assay was performed with the Endosafe Endochrome-K kit (Charles River, no. R1708K), where a control standard endotoxin (CSE) was diluted from 5 to 0.005 EU/mL. Samples were diluted 1:100 and plated in triplicate. An equal volume of LAL was added to each well. A Tecan Spark plate reader (Tecan, Mȁnnendorf, Switzerland, no. 30086376) with SparkControl magellan v.2.2 software was used at 37°C to read absorbance at 405 nm every 30 s for at least 80 cycles. Time at which absorbance crossed an OD of 0.1 was recorded and used to determine endotoxin levels.

The cartridge-based LAL assay was performed using an Endosafe nexgen-PTS machine with R&D cartridges as recommended (Charles River, cat. no. PTS2005, 0.05 EU/mL sensitivity). In brief, samples were diluted 1:50 in a large volume of ET-free water, vortexed, and 25 μL was loaded into each of the four lanes of the cartridge, where two lanes contain CSE spike-in to calculate efficiency of the assay, which is valid from 50% to 200% recovery. The final valid ET value was recorded from the duplicate measurement from a single cartridge.

The PyroGene Recombinant Factor C Endpoint Fluorescent Assay (Lonza, Walkersville, MD, cat. no. 50-658U) was performed as recommended. Kit-supplied CSE was diluted from 5 to 0.005 EU/mL and samples were diluted 1:100 in ET-free water and added to a plate in triplicate. The plate was heated at 37°C for 10 min, then an equal volume of working reagent was added to each well. Fluorescence was read immediately at time 0 and after incubating for 60 min. Relative fluorescence units (RFUs) of the ET-free water only blank wells were subtracted from all measurements, then delta RFUs between the two time points was calculated, and a linear regression was applied to the standard curve to calculate EUs in the samples. Fluorescence measurements were performed on a Cytation5 with Gen 5 3.04 software (BioTek, Winooski, VT).

HEK-Blue cells (hTLR4 and Null2) were purchased from Invivogen (San Diego, CA) and were grown under BSL2 conditions (37°C with 5% CO_2_) to measure SEAP production downstream of NF-κB activation following treatment with Cas9 proteins, guide RNAs, and RNPs *in vitro* as recommended. Cells were grown in T-75 flasks with supplied antibiotic selection markers and passaged at 70% confluency. Cells were detached with gentle scraping in 1× PBS, centrifuged, counted, and plated for experiments in freshly prepared HEK-Blue Detection Medium at approximately 32,000 cells per well in a 96-well plate. Cell suspension (180 μL) was plated directly into 20 μL of diluted CSE (5–0.078 ng) or samples (diluted to 10 μM) and incubated overnight at 37°C. Absorbance was read at 620 nm in a Tecan Spark plate reader.

### NPC line creation and culture

NPCs were isolated from Ai9-tdTomato homozygous mouse embryos (day 13.5) by microdissection of cortical tissues into Hibernate E (#HECA, BrainBits LLC, Springfield, IL) and processing with the Neural Dissociation Kit with papain (Miltenyi, Bergisch Gladbach, Germany, no. 130-092-628) according to the manufacturer’s instructions. Single cells grew into non-adherent neurospheres, which were maintained in culture medium (DMEM/F12 [Thermo Fisher Scientific, Waltham, MA, no. 10565-018], B-27 supplement [no. 12587-010], N-2 supplement [no. 17502-048], MEM non-essential amino acids [no. 11140-050], 10 mM HEPES [no. 15630-080], 1,000× 2-mercaptoethanol [no. 21985-023], 100× Pen/Strep [no. 15140-122]) supplemented with growth factors (FGF-basic [BioLegend, no. 579606] and EGF [Thermo Fisher Scientific, no. PHG0311]) to a final concentration of 20 ng/mL in medium. Neurospheres were passaged every 6 days using the Neural Dissociation Kit to approximately 1.5 million cells per 10-cm dish and growth factors were refreshed every 3 days. Cells were authenticated by immunofluorescent staining for Nestin and GFAP, routinely tested for mycoplasma, and were used for experiments between passages 2 and 20. Dissociated cells were grown in monolayers in 96-well plates pre-coated with poly-DL-ornithine (Sigma-Aldrich, no. P8638), laminin (Sigma-Aldrich, no. 11243217001), and fibronectin (Sigma-Aldrich, no. F4759) (“PLF”) for direct delivery and nucleofection experiments at approximately 30,000 cells per well.

### Human iPSC differentiation into NPCs and culture

MSC-iPSC1 cells were a generous gift from Boston Children’s Hospital. iPSCs were differentiated into NPCs based on dual SMAD inhibition as described previously. In brief, iPSCs were plated onto Matrigel in the presence of 10 μM Y-27632 (Sigma, no. Y0503) at a density of 200,000 cells/cm^2^. The next day (day 0) medium was changed to KSR medium (Knockout DMEM [Thermo Fisher Scientific, no. 10829018], 15% Knockout serum replacement [Thermo Fisher Scientific, no. 10828010], L-glutamine [1 mM], 1% MEM non-essential amino acids, and 0.1 mM β-mercaptoethanol). Medium was changed daily during differentiation and gradually changed from KSR medium to N2/B27 medium (Neurobasal medium [Thermo Fisher Scientific, no. 21103049], GlutaMAX supplement [Thermo Fisher Scientific, no. 35050061], N-2 supplement [Thermo Fisher Scientific, no. 17502048], and B-27 supplement [Thermo Fisher Scientific, no. 17504044]) by increasing N2/B27 medium to 1/3 on day 4, 2/3 on day 6, and full N2/B27 medium on day 8. For the first 12 days of differentiation, the medium was supplemented with 100 nM LDN193189 (Sigma, no. SML0559) and 10 μM SB431542 (Tocris Bioscience, Bristol, England, no. 1614). For the first 4 days, the medium was also supplemented with 2 μM XAV939 (Tocris Bioscience, no. 3748). On day 19, NPCs were dissociated with StemPro Accutase (Thermo Fisher Scientific, no. A1110501) and replated onto Matrigel for expansion. NPCs were passaged every 6 days and maintained in NPC medium (DMEM/F12, N2 supplement, B27 supplement, and 20 ng/mL bFGF [Corning, Corning, NY, no. 354060]) with medium changes every other day. For direct delivery experiments, 12,000 cells were seeded in Matrigel in a 96-well plate and after 48 h were treated in triplicate with 100 pmol of 4xNLS-SpyCas9-2xNLS RNPs with the EMX1 guide RNA (spacer: 5′ GAGTCCGAGCAGAAGAAGAA) or non-targeting guide RNA (spacer: 5′ AACGACTAGTTAGGCGTGTA). In the Lipofectamine CRISPRmax group (Thermo Fisher Scientific, no. CMAX00003), 3 μg of 0xNLS-SpyCas9-2xNLS protein (18 pmol) was mixed with sgRNA (1:1 M ratio) in 8 μL OptiMEM with 6 μL of Cas9 Plus Reagent (1 μg protein:2 μL reagent) and was mixed with a second tube containing 3.6 μL CRISPRmax reagent in 8 μL OptiMEM, incubated for at least 5 min and was immediately distributed to cells in triplicate (1 μg RNP per well) according to the manufacturer’s recommendations.

### Cas9 RNP assembly and delivery to cells

For cell culture experiments, RNPs were prepared immediately before use at a 1.2:1 M ratio of sgRNA (Synthego, Redwood City, CA) to protein (QB3 Macrolab or Aldevron). The solution was incubated for 5–10 min at room temperature. For nucleofection, RNPs were formed at 10 μM in 10 μL of pre-supplemented buffer (Lonza P3 Primary Cell 96-well Kit, no. V4SP-3096). A 15-μL suspension of 250,000 mouse NPCs was mixed with 10 μL RNP solution and added to the nucleofection cuvette. Nucleofection was performed using the 4D Nucleofector X Unit (Lonza, no. AAF-1003X) with pulse code EH-100 and cells were recovered with 75 μL medium per well. Nucleofected cells were then transferred to 100 μL fresh medium in 96-well plates in triplicate and allowed to grow for 5 days at 37°C before analysis by flow cytometry for tdTomato expression. For direct delivery, RNPs were formed at 10 μM in 10 μL of sterile buffer 1 (25 mM sodium phosphate [pH 7.25], 100 mM NaCl, 200 mM trehalose). After NPCs were grown for 2 days in an adherent monolayer, 10 μL was added to cell monolayer (“direct delivery”) for a final concentration of 1 μM (100 pmol RNP/100 μL medium). Medium was changed 48 h post-treatment and cells were collected 5 days post-treatment for analysis by flow cytometry for tdTomato expression or 4 days post-treatment for DNA sequencing.

For *in vivo* experiments, RNPs were prepared similarly at 10 μM concentration in buffer 1 and incubated at 37°C for 10 min. RNPs were sterile filtered by centrifuging through 0.22-μm Spin-X cellulose acetate membranes (Corning CoStar, no. 32119210) at 15,000 × *g* for 1 min at 4°C. RNPs were then concentrated using 100 kDa Ultra-0.5 mL Centrifugal Filter Unit (Amicon, Burlington, MA) at 14,000 × *g* at 4°C until the final desired concentration was reached (25–100 μM, minimum 50 μL volume) and collected by centrifuging at 1,000 × *g* for 2 min. RNPs were then divided into single-use 20 μL aliquots, flash-frozen in liquid nitrogen, and stored at −80°C until the experiment. Prior to intracranial injection, RNPs were thawed, pipetted to mix, loaded into a 25 μL syringe (Hamilton, Reno, NV, no. 7654-01) and injected with custom 29-gauge CED cannulas.

### AAV9 transduction

A single 50-μL aliquot of AAV9-CMV-SauCas9-U6-sgRNA or AAV9-CMV-GFP (Virovek) was thawed from −80°C and stored at 4°C. AAV9 was diluted in 1× PBS without calcium or magnesium to the desired concentration. For *in vivo* experiments, AAV was diluted on the day of surgeries to 3e-8 to 3e-9 vg/μL and stored on ice until loaded into the syringe. Five microliters were injected in each hemisphere to a final dose of 1.5e-9 to 1.5e-10 vg per hemisphere using CED conditions (0.5 μL/min). For cell culture experiments, serial dilutions were performed from 1.6e-9 to 2e-8 vg/μL (lowest MOI = 200,000) and 10 μL of each AAV dilution was added simultaneously with NPCs in PLF-coated 96-well plates in triplicate. Treated cells were maintained for 3–9 days before flow cytometry for GFP expression (transduction) and tdTomato expression (genome editing).

### Empty capsid quantification by cryoelectron microscopy

AAV samples were frozen using FEI Vitrobot Mark IV cooled down to 8°C at 100% humidity. In brief, 4 μL of AAV9 capsids containing GFP or Cas9 cargo was deposited on 2/2 400 mesh C-flat grids (Electron Microscopy Sciences, Hatfield, PA, no. CF224C-50), which were previously glow discharged at 15 mA for 15 s on PELCO easyGLOW instrument. Grids were blotted for 3 s with blot force 8 and wait time 2.5 s. Micrographs were collected manually on Talos Arctica operated at 200 kV and magnification 36,000× (pixel size 1.14 Å/pix) using a super-resolution camera setting (0.57 Å/pix) on K3 Direct Electron Detector. Micrographs were collected using SerialEM v.3.8.7 software. Capsids were counted manually by three blinded reviewers for each image and the three counts were averaged and reported as percentage empty capsids between the two groups.

### RNP size and aggregation measurement by dynamic light scattering

RNPs were prepared at 25 μM, according to the methods described above for *in vivo* experiments. Approximately 40 μL of RNP was added to a disposable cuvette (ZEN0040) and inserted into the Zetasizer Nano (ZSP, Malvern, UK) then 173° scattering angle was measured at 25°C for 5 min or 37°C after incubation for 30 min. Size distribution by mass histograms are shown with estimated peak size in nanometers (nm). Each sample was analyzed three times, resulting in a histogram generated from 10 individual measurements.

### Analysis of editing *in vitro*

tdTomato positivity was assessed by flow cytometry using IGI facilities on the Attune NxT (Thermo Fisher Scientific, AFC2). In brief, mouse NPCs were washed once with 1× PBS, harvested with 0.25% trypsin, neutralized with DMEM containing 10% FBS, and resuspended in 150 μL of 1× PBS per well of a round-bottom 96-well plate for analysis. The percentage of tdTomato^+^ cells from each well was recorded. For analysis of gDNA, medium was removed, cells were rinsed once with 1× PBS, then incubated with 100 μL QuickExtract solution (Lucigen Corporation Supplier Diversity Partner QuickExtract DNA Extraction Solution 1.0, Thermo Fisher Scientific, no. QE09050) at 37°C for 5 min. The cell lysate was then moved to a thermal cycler and incubated at 65°C for 20 min and 95°C for 20 min. gDNA was used in PCR reactions to generate amplicons of approximately 150–300 bp for Illumina sequencing. A list of primers used for NGS is provided in Table S1. Sequencing was performed with Illumina MiSeq in the IGI Center for Translational Genomics and reads were analyzed in CRISPResso2 (http://crispresso.pinellolab.org/submission).^61^

### Stereotaxic infusion of Cas9 RNPs and AAVs

Ai9 mice (Jackson Laboratory, Bar Harbor, ME, no. 007909) were group housed at the University of California, Berkeley, with a 12-h light-dark cycle and allowed to feed and drink *ad libitum.* Housing, maintenance, and experimentation of the mice were carried out with strict adherence to ethical regulations set forth by the Animal Care and Use Committee (ACUC) at the University of California, Berkeley. Cas9-RNP and AAVs were prepared on-site at the University of California, Berkeley, for injection into male and female tdTomato Ai9 mice aged between 2 and 5 months. All tools were autoclaved and injected materials were sterile. Mice were anesthetized with 2% isoflurane, given pre-emptive analgesics, and arranged on an Angle Two Stereotactic Frame (Leica, Nussloch, Germany). The incision area was swabbed with three alternating wipes of 70% ethanol and betadine scrub with sterile applicators before performing minimally damaging craniotomies. The stereotaxic surgery coordinates used for targeting the striatum, relative to bregma, were +0.74 mm anteroposterior, ±1.90 mm mediolateral, −3.37 mm dorsoventral. Bilateral CED infusion of Cas9 RNPs (10–100 μM) or Cas9 AAVs (3e-8 to 3e-9 vg/μL) was performed with a syringe pump to deliver 5 μL at 0.5 μL per minute (Model 310 Plus, KD Scientific, Holliston, MA) with a step or non-step cannula. For i.c.v. infusion of Cas9 RNPs, cannulas were placed at −0.7 mm anteroposterior, ±1.2 mm mediolateral, and −2.5 mm dorsoventral according to the Paxinos atlas of the adult mouse. Post-infusion, the syringes were left in position for 2 min before slow removal from the injection site, which was then cleaned, sutured, and surgically glued. Throughout the procedure, mice were kept at 37°C for warmth and Puralube Vet Ointment (Dechra, Northwich, England, NDC no. 17033-211-38) was applied to the outside of the eyes. For i.c.v. injection of P0 neonatal mice, anesthesia was induced by hypothermia, then 4 μL of 100 μM Cas9-RNP was injected with a handheld 33-gauge needle unilaterally with 10% Fast Green dye to visualize distribution from one ventricle throughout the CNS. The needle was inserted 2 mm deep at a location approximately 0.25 mm lateral to the sagittal suture and 0.50–0.75 mm rostral to the neonatal coronal suture. RNP was slowly injected, then the needle was held in place for 15 s, and mice were monitored until recovery. For i.t. injection, anesthetized mice received a 5-, 25-, or 50-μL bolus injection of Cas9 RNP at 300 μM. The 29-gauge needle was inserted at the L6-S1 vertebral junction and angled slightly rostrally for the injection. Mice were allowed to fully recover before being transferred back to their housing. Recovery weight following all procedures was monitored daily for 1 week and mice were housed without further disruption for various time periods until tissue collection.

### Tissue collection and immunostaining

At the defined study endpoints (3, 21, and 90 days post-injection), mice were placed under anesthesia and tissues were perfused with 10 mL of cold PBS and 5 mL of 4% paraformaldehyde (PFA) (Electron Microscopy Sciences, no. 15710). Brains were post-fixed overnight in 4% PFA at 4°C, rinsed 3× with PBS, then cryoprotected in 10% sucrose in PBS solution for approximately 3 days. Brains were embedded in optimal cutting temperature medium (Thermo Fisher Scientific, no. 23-730-571) and stored at −80°C. Brains were cut at 20–35-μm-thick sections using a cryostat (Leica CM3050S) and transferred to positively charged microscope slides. For immunohistochemical analysis, tissues were blocked with solution (0.3% Triton X-100, 1% bovine serum albumin [Sigma-Aldrich, no. A9418], 5% normal goat serum [Sigma-Aldrich, no. G9023]) before 4°C incubation overnight with primary antibody in blocking solution. The next day, tissues were washed three times with PBS and incubated with secondary antibodies for 1 h at room temperature. After three PBS washes, samples were incubated with DAPI solution (0.5 μg/mL, Roche LifeScience, Penzberg, Germany) as a DNA fluorescence probe for 10 min, washed three times with PBS, submerged once in deionized water, and mounted with glass coverslips in Fluoromount-G slide mounting medium (SouthernBiotech, Birmingham, AL). Primary antibodies included rabbit polyclonal anti-S100β (1:500, Abcam, Cambridge, England, no. ab41548), rabbit polyclonal anti-Olig-2 (1:250, Millipore Sigma, Burlington, MA, no. AB9610), rabbit polyclonal anti-doublecortin (1:800, Cell Signaling Technology, Danvers, MA, no. 4604), rabbit polyclonal anti-Ki67 (1:100, Abcam, no. ab15580), mouse monoclonal anti-NeuN (1:500, Millipore Sigma, no. MAB377), rabbit polyclonal anti-DARPP-32 (1:100, Cell Signaling Technology, no. 2302), rabbit polyclonal anti-Iba1 (1:100, Wako Chemicals, Richmond, VA, no. 019–19741), mouse monoclonal anti-glial fibrillary acidic protein (1:1,000, Millipore Sigma, no. MAB3402), rat monoclonal anti-CD45 (1:200, Thermo Fisher Scientific, no. RA3-6B2), and rabbit polyclonal anti-CD3 (1:150, Abcam, no. ab5690). Secondary antibodies included donkey anti-rat 488 (1:500, Thermo Fisher Scientific, no. A-21208), goat anti-rabbit 488 (1:500, Thermo Fisher Scientific, no. A32731), goat anti-rabbit 647 (1:500, Thermo Fisher Scientific, no. A21245), and goat anti-mouse IgG1 647 (1:500, Thermo Fisher Scientific, no. A-21240).

### Fluorescent imaging and image quantification

Whole-brain sections were imaged and stitched using the automated AxioScanZ1 (Zeiss, Oberkochen, Germany) with a 20× objective in the DAPI and tdTomato channels. Images generated from slide scanning were viewed in ZenLite software (v.3.6 blue edition) as CZI files. Images were then exported to FIJI (v.1.53q), Imaris (v.9.9.1), or QuPath (v.0.3.2) for quantification by authors blinded to the sample identity. The area of reflux from CED needles was calculated directly in ZenLite using the shape and area analysis tools. Immunostained cells and tissues were imaged on the Evos Revolve widefield microscope using a 20× objective or Stellaris 5 confocal microscope (Leica) with a 10× or 25× water immersion objective to capture data in DAPI, FITC, tdTomato, and CY5 channels. Approximately four images were taken at 20–25× per hemisphere across multiple sections for image quantification of CD45, Iba1, and CD3 (8–12 images quantified and averaged per injection). Approximately four to six z stack images were captured and stitched per hemisphere for qualitative images of Iba1 and for quantification of NeuN, DARPP-32, ALDH1L1, and OLIG2 with tdTomato at 1,024 × 1,024 pixel resolution with a scanning speed of 100–200.

Measurements of striatal editing by volume were conducted using QuPath software (v.0.3.2) from images obtained from the Zeiss AxioscanZ1. In brief, ROIs were drawn to outline the border of each striatum and the inner area of tdTomato editing using the polygon tool to create annotations. All coronal plane areas were automatically calculated. Dorsoventral coordinates (relative to bregma) were then estimated in millimeters by consulting the Mouse Brain Atlas (C57BL/6J Coronal). Approximate tissue volume was calculated by averaging outlined areas between consecutive sections to represent the mean area across a dorsoventral segment and multiplying by the difference in dorsoventral coordinates. Edited striatal volumes were then divided by total striatal volumes to obtain percent editing. Additional tdTomato^+^ cell count measurements were conducted in Imaris software v.10.0 (Oxford Instruments, Abingdon, UK). In brief, ROIs were drawn over each hemisphere (including cells in all brain sub-structures) using the “Segment only a Region of Interest” tool, and positive cells detected using the automated “Spots” tool to provide cell counts. Thresholds were adjusted manually. Counts of tdTomato cells on each image were then related back to approximate coordinates relative to bregma using the Mouse Brain Atlas (C57BL/6J Coronal) to quantify the distribution of edited cells.

Cell-type-specific measurements were conducted using QuPath software (v.0.3.2) on images obtained from Stellaris 5 z stack maximal projections. ROIs were again drawn around areas of observed tdTomato editing, using the polygon tool to create a single annotation per image. Cell count calculations were performed using the “Cell Detection” and “Positive Cell Detection” tools, adjusting “Cell Mean” thresholds accordingly for each channel and image. Percent area and intensity measurements were performed in FIJI/ImageJ software (v.2.1.0/1.53c). Images were converted to 32-bit, and thresholds were adjusted to detect the corresponding stained area. Measurements were set to include area, minimum, maximum, and mean gray value, and area fraction, as well as to limit to threshold. All image quantification was performed on two to five serial sections with three to ten independent injections per group for each analysis. Cell counts, area, intensity, and volume measurements were in general averaged from multiple sections and then grouped with other biological and technical replicates, to report the treatment group average with standard deviation displayed by bar graph or box and whisker plot.

### Serum collection and ELISA

Blood was collected from mice at the time of euthanasia, allowed to clot at room temperature for 15–30 min, then centrifuged for 5 min at 2,000 × *g*. Serum was collected and placed immediately on dry ice and then stored at −80°C. Enzyme-linked immunosorbent assays were performed using the SeraCare Protein Detector HRP Microwell Anti-Mouse ELISA Kit, no. 5110-0011 (54-62-18) according to the manufacturer’s recommendations. First, 96-well plates were coated with antigens of interest (0.5 μg protein per well for SauCas9 and SpyCas9 (4xNLS protein variants) and approximately 1e-9 empty AAV capsids per well) overnight at 4°C. Wells were washed three times and blocked at room temperature for 1 h. Serum samples were then incubated in wells at varying concentrations (1:50 to 1:10,000 dilution) in 1× blocking buffer for 4 h at room temperature, along with monoclonal antibody controls to generate standard curves. Standards included CRISPR-Cas9 Monoclonal Antibody 7A9 (Epigentek, Farmingdale, NY, no. A-9000-050), GenCRISPR SaCas9 Antibody 26H10 (GenScript, Piscataway, NJ, no. A01952), and Anti-Adeno-associated Virus 9 Antibody clone HL2374 (Millipore Sigma, no. MABF2326-25UG). Following three additional washes, the HRP secondary antibody was added at 1:500 in 1× blocking buffer and incubated for 1 h. Wells were then washed three more times, and peroxidase substrate solutions were added. Absorbance was recorded at a wavelength of 405 nm with Cytation5 plate reader with Gen 5 3.04 software (BioTek). Serum antibody concentrations were calculated using four-parameter logistic curve data analysis (4PL, MyAssays.com) and normalized to sham controls.

### Splenocyte collection and ELISpot

Spleens were collected at the time of euthanasia and stored in medium composed of RPMI 1640 (Thermo Fisher Scientific, no. 11875-119) with 10% FBS (VWR, no. 89510-186, Radnor, PA) and 1% Pen/Strep (Thermo Fisher Scientific, no. 15140-122). In brief, spleens were physically dissociated with a 100-μm cell strainer in 10 mL of medium then single cells were passed through a 70-μm strainer and centrifuged at 200 × *g* for 5 min. Cells were resuspended in 5 mL of 1× RBC Lysis Buffer (Miltenyi, no. 130-094-183) for approximately 3 min, then centrifuged again and resuspended in medium for counting. The mouse IFN-γ ELISpot kit (R&D Systems, Minneapolis, MN, no. EL485) was used according to the manufacturer’s instructions to assess activation of splenocytes, containing T cells, in response to treatment with Cas9 proteins. In brief, the plate was pre-incubated with 200 μL of medium for at least 20 min, before adding cells at 300,000 per well in 100 μL medium. Treatments at 2× dose were prepared in medium and 100 μL was added to wells in triplicate (final 5 μg/mL concentration). Plates were incubated for 48 h without disturbing. Concanavalin A (Sigma-Aldrich, no. C5275) was used as a positive control for cell-mediated IFN-γ production (final 4 μg/mL concentration). After 48 h, cells were removed and the secreted analyte was detected with immunostaining using the kit-provided biotinylated monoclonal antibody specific for mouse IFN-γ, streptavidin-conjugated alkaline phosphatase, and stabilized detection mixture of 5-bromo-4-chloro-3′-indolylphosphate-p-toluidine salt and nitro blue tetrazolium chloride. After staining, plates were dried overnight and spot forming units were imaged and counted on the ImmunoSpot S6 Macro Analyzer (Cellular Technology Limited, Shaker Heights, OH).

### Pre-treatment of mice with Cas9 and adjuvant

AddaVax (Invivogen, vac-adx-10), a squalene-based oil-in-water nano-emulsion, was mixed with an equal volume containing 25 μg of 4x-SpyCas9-2x protein diluted in sterile buffer at room temperature for a final injection volume of 50 μL. Mice received two 25 μL subcutaneous injections of the AddaVax:Cas9 mixture (immunized) or AddaVax:Buffer alone (sham) with a 30-gauge insulin syringe into each flank. After 4 weeks, stereotaxic surgery with bilateral injections of 5 μL of 25 μM Cas9-RNPs was performed in a subset of the mice. Mice showed no signs of pain or distress following treatment with AddaVax and no acute events were noted after surgery. Mice that received AddaVax, with or without surgery, were euthanized 6 weeks post-subcutaneous injections. Brains, serum, and spleens were collected for analysis of adaptive immune responses against repeated exposure to Cas9.

### DNA/RNA extraction from brain tissue slices and qRT-PCR, ddPCR, and long-read sequencing

Brains were collected at 3, 28, or 120 days (4 months) for DNA and RNA analysis. In brief, mice were placed under anesthesia and perfused with cold PBS. Brains were harvested and cut into 2-mm sections using a matrix around the injection site (Zivic Instruments, Pittsburgh, PA). The slices were transferred onto chilled glass slides and further trimmed to approximate 30 mg tissue weight (1–1.25 mm wide × 2 mm long). Tissues were flash frozen in liquid nitrogen then stored at −80°C until processing. DNA and RNA were collected from tissues using the AllPrep DNA/RNA Mini Kit (QIAGEN, Venlo, the Netherlands, no. 80204) according to the manufacturer’s instructions. In brief, brains were homogenized in 1.5-mL tubes with a disposable pestle directly in RLT lysis buffer supplemented with 2-mercaptoethanol, then passed through Qiashredder columns before adding directly to the DNA and RNA binding columns. DNA was eluted in 100 μL of EB and RNA was eluted in 40 μL RNAse-free water. Concentrations of nucleic acids were measured using a NanoDrop spectrophotometer and samples were stored at −20°C.

Gene expression was quantified across multiple samples using a Custom RT^2^ PCR Array (QIAGEN, no. 330171, CLAM45824) and analyzed using the RT^2^ Profiler PCR Data Analysis Tool on GeneGlobe (QIAGEN). For reverse transcription, the RT^2^ First Strand Kit (QIAGEN, no. 330404) was used according to the manufacturer’s instructions. cDNA was diluted in water and added to the RT^2^ SYBR Green qPCR Mastermix (QIAGEN, no. 330502) then distributed across the 24-wells containing verified assay primers and controls (PCR array reproducibility control, reverse transcription efficiency control, gDNA contamination control, two housekeeping genes, and 19 experimental genes). Quantitative real-time PCR was performed on the CFX96 Touch Real-Time PCR System (Bio-Rad). cDNA was also used in a ddPCR reaction to measure SauCas9 expression at the 4-month time point. qPCR assay IDs are included in Tables S1 and S2.

Off-target sites were predicted using Cas-OFFinder (http://www.rgenome.net/cas-offinder/),^62^ described further in Tables S3 and S4. Primers were designed using NCBI Primer Blast with an amplicon size of 250–300 bp, listed in Table S1. Sequencing was performed with Illumina MiSeq in the IGI Center for Translational Genomics and reads were analyzed in CRISPResso2 (http://crispresso.pinellolab.org).^61^

For ddPCR, custom NHEJ ddPCR assays were generated using the online Bio-Rad design tool (Table S2). Assays for SauCas9 and SpyCas9 contain both the primers and probes (HEX-probe spanning the cut-site and a distal reference FAM-probe). To prepare the reactions, 110 ng of gDNA was combined with the 20× assay, 2× ddPCR Supermix for Probes (No dUTP), 1 μL of SmaI restriction enzyme (2 units per reaction), and water up to 22 μL. Then 20 μL of each reaction mix was added to DG8TM Cartridges (Bio-Rad, Hercules, CA, no. 1864008) followed by 70 μL of Droplet Generation Oil for Probes (Bio-Rad, no. 1863005) and droplets were formed in the QX200 Droplet Generator. Droplets were then transferred to a 96-well plate and thermal cycled according to the manufacturer’s recommendation with a 3-min annealing/extension step. After thermal cycling, the sealed plate was placed in the QX200 Droplet Reader and data were acquired and analyzed in the QuantaSoft Analysis Pro Software using the “Drop-Off” analysis, manually setting the thresholds for cluster calling (FAM+ only, FAM+ HEX+ cluster, FAM–HEX– cluster), and exporting fractional abundance calculations.

Long-read sequencing of the tdTomato locus was performed on DNA isolated from the treated mouse brains. In brief, PCR amplicons were generated on samples from the 4-month treatment groups using primers with unique barcodes for sample de-multiplexing. The KAPA HiFi Hotstart PCR Kit (Roche, KK2502) was used to amplify the 1,100-bp product and reactions were cleaned with AMPure XP magnetic beads (Beckman Coulter, Brea, CA) before analysis by Qubit and Bioanalyzer with the DNA 7500 Kit (Agilent, Santa Clara, CA, no. 5067-1506). Samples were combined with 1 μg of pooled amplicons and submitted (>20 ng/μL) for sequencing with one PacBio Sequel 8M SMRT Cell at the QB3 Vincent Coates Genomic Sequencing Lab, yielding approximately 110,000 reads per sample. Data were analyzed using a custom pipeline to identify viral fragment trapping during DNA repair. In brief, PacBio circular consensus reads were trimmed with Cutadapt (v,4.1),^63^ then aligned to the AAV vector using NGMLR (v,0.2.7)^64^ to generate BAM files. Soft-clipped regions of aligned reads were extracted using PySam (v,0.18.0, https://github.com/pysam-developers/pysam) to parse CIGAR strings, then realigned to the tdTomato locus with NGMLR to verify integration within 200 bp of the cut site. Confirmed integrations were visualized along the AAV genome using pyGenomeTracks (v.3.3) and coverage statistics were summarized using PySam.^65^^,^^66^

### Statistical analyses

The data presented in bar graphs and box and whisker plots are averages across multiple technical and biological replicates and error bars represent the standard deviation. Sample sizes are indicated in the text and figure legends (generally bilateral injections were treated independently, i.e., two technical replicates per one biological replicate). When comparing two groups with normal distribution, an unpaired Student’s t test was performed in Prism 9 (GraphPad Software v.9.4.1). When comparing multiple groups, a one-way ANOVA with Tukey’s multiple comparison test was performed in Prism 9 (GraphPad Software v.9.4.1). The qRT-PCR experiments used Student’s t test of the experimental group compared with the sham control (QIAGEN GeneGlobe RT^2^ Profiler PCR Data Analysis). p ≤ 0.05 was considered significant.

## Data Availability

Long-read sequencing (BAM files from PacBio circular consensus sequence) are available in Sequence Read Archive: PRJNA986952. All additional data are available upon request.

## Supplemental information

Document S1. Figures S1–S14 and Tables S1–S4

Document S2. Article plus supplemental information
